# Degenerated nerve grafts provide similar quality and outcome in reconstructing critical nerve defects as compared to fresh nerve grafts

**DOI:** 10.3389/fcell.2025.1568935

**Published:** 2025-06-03

**Authors:** Philipp Tratnig-Frankl, Martin Schmoll, Udo Maierhofer, Johanna Klepetko, Florian J. Jaklin, Lisa H. Jöns, Homayon Zirak, Christopher Festin, Leopold Harnoncourt, Vlad Tereshenko, Konstantin D. Bergmeister, Oskar C. Aszmann

**Affiliations:** ^1^ Clinical Laboratory for Bionic Extremity Reconstruction, Department of Plastic, Reconstructive and Aesthetic Surgery, Medical University of Vienna, Vienna, Austria; ^2^ Center for Medical Physics and Biomedical Engineering, Medical University of Vienna, Vienna, Austria; ^3^ Clinical Department of Plastic, Aesthetic and Reconstructive Surgery, University Clinic of St. Poelten, St. Poelten, Austria; ^4^ Department of Plastic, Reconstructive and Aesthetic Surgery, Medical University of Vienna, Vienna, Austria

**Keywords:** plexus injuries, plexopathy, plexus reconstruction, degenerated nerve grafts, nerve reconstruction

## Abstract

**Introduction:**

Brachial plexus injuries are commonly caused by stretch-traction injuries. The clinical standard is timely anatomic reconstruction with autologous nerve grafts and/or intra- or extraplexal nerve transfers. Commonly used nerve grafts are the sural nerves and/or grafts taken from the affected side. If the lower trunk has been affected, the latter nerves, however, are predegenerated. In this animal experiment we investigated, whether a degenerated nerve graft avails the same quality of regeneration as compared to a non-degenerated graft.

**Methods and materials:**

In this animal study, a 2 cm lesion of the right common peroneal nerve was created, and the ipsilateral sural nerve was cut or left intact to later serve as a graft. Nerve reconstruction was carried out 3 weeks later using the fresh or degenerated graft. After 6 weeks, either a retrograde labeling of the common peroneal nerve or muscle force testing was performed.

**Results:**

A total of 34 male SD rats, Group A (*n* = 13) and Group B (*n* = 21) were included. In Group A, the retrograde labeling of the spinal motor neurons showed an average of 66.05 (±17.03) neurons in animals with a fresh graft and 41.19 (±10.47) neurons in animals with a degenerated graft. In two animals with a fresh graft, no motor neurons could be labeled. No statistical inferiority was observed (*p* = 0.071). In Group B, regeneration is expressed as a recovery ratio. The fresh graft group had a mean maximum evoked contraction of 8.2 (±7.1), compared to 8.5 (±4.9) in the degenerated graft group (*p* = 0.462). The mean maximum twitch force was 5.2 (±3.5) and 6.4 (±4.4) respectively (*p* = 0.577). The mean muscle weight, comparing injured to uninjured side, was 0.32 (±0.06) in the fresh graft group and 0.32 (±0.04) in the degenerated graft group (*p* = 0.964).

**Conclusion:**

The use of predegenerated nerve grafts for critical nerve reconstruction showed no statistical inferiority as compared to the fresh grafts in any of the evaluated outcome. Overall, these results are promising, particularly in the context of critical nerve defects involving multiple nerves, where the use of a degenerated grafts often remains the only additional source of graft material.

## 1 Introduction

The incidence of complex nerve injuries, particularly those involving the brachial plexus, are largely due to high-energy traumas associated with motorcycle accidents, with a disproportionately high rate among young adults, especially males, who are at greater risk of traumatic injuries (Moran et al., 2005; Leonard et al., 2020; Fogel et al., 2021; Shah et al., 2021; Kim et al., 2004; Zaidman et al., 2024). Given the young demographic, these injuries have far-reaching socioeconomic consequences, as they frequently occur during prime working years (Kim et al., 2004; Zaidman et al., 2024).

Similar to peripheral nerve injuries, nerve repair in the brachial plexus should be attempted with the primary reconstructive goal of restoring function and strength (Bengtson et al., 2008). Despite advancements in surgical techniques, a significant portion of these injuries, particularly complex or pan-plexopathies, do not achieve satisfactory results (Kretschmer et al., 2009). Many patients experience lasting impairments in shoulder, arm and hand function, affecting essential tasks and leading to chronic disability with a substantial number of patients unable to return to work (Moran et al., 2005; Brown et al., 2023; Midha, 1997; Noland et al., 2019).

In addition to traumatic injuries in adults, brachial plexus injuries can result from obstetric trauma during complicated childbirth, leading to lifelong functional impairments in infants (Pondaag and Malessy, 2021). Furthermore, non-traumatic conditions, such as tumors compressing or infiltrating the brachial plexus, also contribute to these complex injuries (Rubin, 2020). Another cause of complex brachial plexus injuries is iatrogenic injury, which can occur unintentionally during neck, or chest surgeries, especially first rib or tumor resection (Dengler et al., 2017). These injuries may result from direct trauma or prolonged nerve compression due to positioning (Dengler et al., 2017). Iatrogenic brachial plexus injuries are especially challenging to treat, as they often involve multiple nerve branches and may not be immediately recognized, delaying intervention and potentially reducing the chances for successful recovery (Dengler et al., 2017).

Depending on the complexity of the lesion, if primary tension-free nerve repair is not feasible, using an autologous or allogenic nerve graft is considered the clinical gold-standard before nerve, tendon or free muscle transfers should be performed (Fox and Mackinnon, 2011; McQuillan et al., 2024; Tung and Mackinnon, 2010). It is generally recommended, to perform reconstruction within 6 months to obtain the optimal clinical result (Martin et al., 2019).

The most commonly used nerve grafts are the sural nerve and the medial antebrachial cutaneous nerve (Norkus et al., 2005; Masear et al., 1989). However, procuring one of these nerves comes with donor-side morbidities, such as loss of sensation or neuroma formation (Fox and Mackinnon, 2011). Following trauma distal to the site of injury, the degeneration of nervous structures commences rapidly, typically within 24–48 h after injury. This degeneration process, known as Wallerian degeneration, continues over the following days to weeks, as the axons and myelin sheaths break down and are gradually cleared by macrophages and reactive Schwann cells (Tomita et al., 2009; Sulaiman and Gordon, 2013; Gaudet et al., 2011).

While significant progress has been made in developing alternatives to autologous nerve grafts, each approach has limitations that affect its practical use. Allografts and decellularized nerve grafts, may still not fully replicate the structure and functionality of native nerves, especially considering long nerve defects (Isaacs et al., 2023; Saffari et al., 2024; Choi et al., 2024). Synthetic and bioengineered conduits, although useful in small nerve gaps, often lack the biological complexity required for larger and longer injuries, and their long-term efficacy in complex cases remains under investigation (Crook et al., 2024). Stem cell-seeded grafts and scaffolds show promise in preclinical studies but face challenges in scalability or consistent cell survival (Yi et al., 2020).

Given these limitations, research into using pre-degenerated autologous nerve grafts offers a potentially valuable alternative by leveraging the body’s tissue, preconditioned by injury, as a more biologically compatible solution for reconstructive nerve surgery in complex cases. In this animal experiment we have investigated the question whether a predegenerated nerve graft avails the same quality of regeneration as compared to a non-degenerate graft, such as a freshly harvested sural nerve.

## 2 Materials and methods

### 2.1 Animals

All experiments were approved by the institutional Committee for Animal Experimentation and the Austrian Federal Ministry of Education, Science and Research (BMBWF, 2022-0.711.027).

Overall, 34 male Sprague Dawley (SD) rats, with a mean weight of 478 (323–600) g, aged 8–12 weeks were enrolled and assigned into two groups (Group A and Group B). Every group included experimental and control animals. Animals had unrestricted access to food and water. Anesthesia was maintained with 1.5% isoflurane following endotracheal intubation.

All animals received piritramide (0.3 mg/kg of body weight) subcutaneously and postoperatively followed by 3 days of drinking water with glucose and piritramide (30 mg piritramide, 30 mL 10% glucose, and 250 mL water). Furthermore, the animals were examined daily for any signs of postoperative distress or surgical infections.

### 2.2 Surgical procedure

**FIGURE 1:**
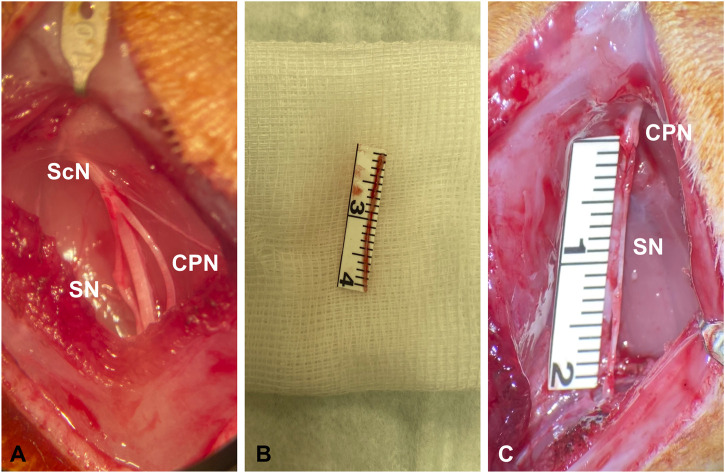
Surgical exposure of the trifurcation of the ScN with the SN and the CPN **(A)**. Harvest of a 2 cm graft of the SN **(B)**. Reconstruction of the CPN with either a fresh or degenerated SN graft **(C)**.

**FIGURE 2:**
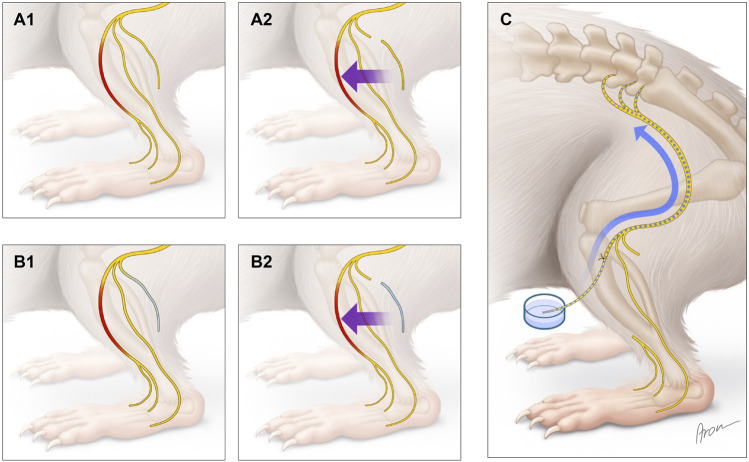
Surgical procedures and retrograde labeling. Creation of a 2 cm CPN defect, leaving the SN intact **(A1)** or cutting the SN (highlighted in blue) **(B1)**. Reconstruction of the CPN using a fresh **(A2)** or degenerated **(B2)** SN graft. Image **(C)** shows the retrograde labeling at follow-up.

Surgical procedures were performed by a single surgeon, using a surgical microscope. Following anesthesia, animals were placed in a prone position, the right lower limb was abducted and prepared in a sterile manner. In a first procedure the gluteal muscle was split, carrying out dissection to the trifurcation of the sciatic nerve (ScN) and its peripheral nerve endings (Figure 1A). Then, a 2 cm defect of the right common peroneal nerve (CPN) was created (Figure 2). In the control animals, the ipsilateral sural nerve (SN) was left intact (Figure 2A1), while in the experimental animals, the SN was cut distal to the trifurcation and left *in vivo* to degenerate (Figure 2B1). Then, layer-wise wound closure was carried out.

After a follow-up of 3 weeks, the CPN was reconstructed either using the fresh SN graft (control) (Figure 2A2) or the degenerated SN graft (experimental) (Figure 2B2). Nerve coaptation was performed with two interrupted 11-0 nylon sutures proximally and distally to avoid rotation of the graft (Figures 1B,C). Six weeks later, in Group A the CPN was retrogradely labeled (Figure 2C), and in Group B, bilateral muscle force testing of the anterior tibialis anterior muscle (TA) was carried out.

### 2.3 Retrograde labeling

Six weeks after nerve reconstruction, in Group A, retrograde labeling of the CPN was performed to visualize spinal motor neurons as described in similar work (Hayashi et al., 2007; Novikova et al., 1997; Bergmeister et al., 2019). Therefore, the CPN was transected distally to the distal nerve coaptation and placed in a reservoir filled with 7 µL of 10% Fluoro Ruby (Invitrogen, Carlsbad, CA) (FR). The nerve stump was left in place for 1 h and kept moisture during this time with Vaseline (Fagron, Germany). Then, the nerve was left *in situ*, the wound closed, and the animal was allowed to recover for another 6–9 days. Subsequently, all animals were anesthetized again with ketamine (200 mg/kg of body weight) and xylazine (5 mg/kg of body weight). After ensuring deep anesthesia, intracardial perfusion through the left cardiac ventricle was performed using 400 mL of 0.9% NaCl followed by 400 mL of 4% paraformaldehyde (PFA). Next, the spinal cord was procured and transferred into 4% PFA for 24 h and stored light-protected at 4°C.

Then, the samples were transferred into phosphate-buffered saline (PBS) for another 24 h before dehydration using increasing glucose solutions (10%, 25% and 40%) dissolved in PBS for 24 h each before embedding and storing at −80°C.

All samples were cut into 40 µm thick longitudinal sections using a cryostat (Leica Microsystems, Germany) before motor neuron count was carried out on the TissueFAXs slide scanner (TissueGnostics, Austria). Nucleus counts were performed using the modified Abercrombie formula (Abercrombie, 1946).

### 2.4 Muscle force testing

To investigate the functionality of the TA muscle, forces during maximum evoked contracture (MEC) and the maximum twitch (MT) were measured bilateral 6 weeks after nerve reconstruction. All animals in Group B were anesthetized, and the CPN including both coaptation sides, was dissected again carefully. The animals were positioned in a supine position before the TA muscle in its total length was exposed and the muscle retinaculum and the muscle tendon cut distally. To restrain the leg of the rat, a 1.5 mm K-wire was drilled trough the femur condyles and both ends of the K-wire fixed into a metal frame. The distal tendon of the TA, a 3–0 silk suture was used to fixate the tendon via a clove hitch. Additional drops of super glue (UHU GmbH & Co. KG, Bühl, Germany) were used to secure the knot. The other end of the suture was attached to a force transducer (KD45 5N, ME Messsysteme, Henningsdorf, Germany), while two needle electrodes for bipolar EMG recordings were positioned longitudinally in the TA.

Once the preparation was finished, electrical stimulation was carried out through a bipolar stainless-steel hook electrode using a customized stimulator (MiniVStim 18B, CTID, Center for Medical Physics and Biomedical Engineering, Medical University of Vienna) delivering pseudo-monophasic pulses (2 mA, 400 μs) with exponential charge-balancing phases. The hook electrode was positioned proximally to the nerve coaptation. Data recording was done at a sample rate of 100 kS/s using a PowerLab 16/35 (ADInstruments, Sydney, Australia). For the muscle force testing, the isoflurane dose was reduced temporarily to 1% to minimize interferences. Force measurements were performed during MEC and MT. MEC’s were elicited at supra-maximal stimulation (A = 2 mA, PhW = 400 μs, F = 40 Hz, 330 ms duration), with 30 s pause in between each test. In order to test MT, three supra-maximal single pulses (A = 2 mA, PhW = 400 μs) were delivered to the nerve with a one second pause in-between each impulse. Between the MEC and the MT testing, a 3-min pause was obtained to ensure muscle recovery.

This procedure was performed on the operated (experimental) and the uninjured (control) side of each animal in this group. Once the measurements were done, all animals were euthanized with an intracardial injection of 1 mL of pentobarbital, following bilateral procurement of the TA and the CPN.

### 2.5 Statistical analysis and data analysis

A comparative analysis was conducted between experimental groups. For statistical analyses SPSS (IBM SPSS Statistics for Macintosh, Version 29.0) and MATLAB (R2010a, The MathWorks Inc., United States) were used. The Kolmogorov-Smirnov test for normal distribution was performed before performing a Levene’s Test for equal variance and *t*-test for group comparison.

For each force recording (Group B), the baseline force (approximately 50 ms) was subtracted to isolate the active absolute muscle force. For both, MEC and MT, the average maximum force from three consecutive measurements was analyzed.

In the EMG recordings, the peak-to-peak (PTP) amplitude was measured for the first stimulation impulse. Similar to the force recordings, the average PTP amplitude from three consecutive measurements was used for analysis of the MEC and MT.

To evaluate regained functionality, a recovery index (RI) was calculated for each animal as demonstrated below (Equation 1). This index was derived by normalizing the peak forces elicited by stimulation of the right CPN relative to the peak forces generated by stimulation of the left CPN (used as the reference). This normalization ensured comparability across animals, accounting for individual variations.

$$RI=\frac{\max\left(F_{active\_experimental}\right)}{\max\left(F_{active\_native}\right)}\;x100$$ (1)

Equation 1: Calculation of the Recovery Index using the left (control) and right (experimental) side of each animal.

## 3 Results

### 3.1 Spinal motor neuron count

**FIGURE 3:**
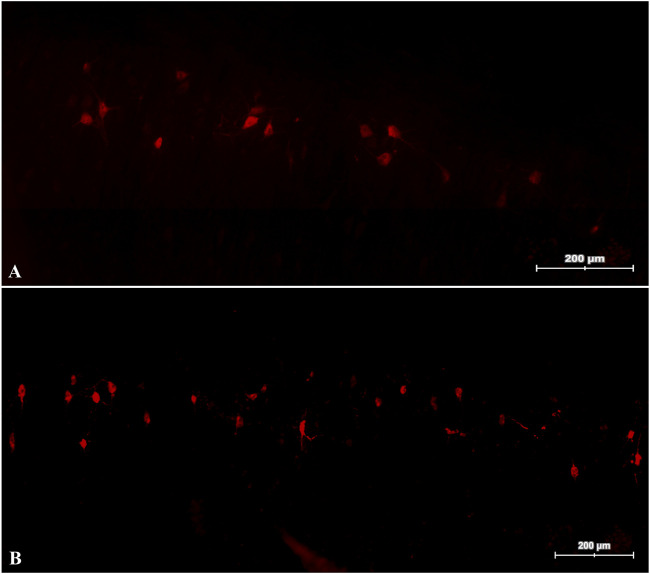
Retrograde labeled spinal motor neurons. Slides of the spinal cord of a rat reconstructed with a fresh **(A)** and a degenerated **(B)** nerve graft.

A total of 13 animals were included (Table 1). Two animals in the control group did not show any spinal motor neurons and have not been included in the final analysis. In the other animals, we counted 66.05 (±17.03) motor neurons in the spinal cord using the fresh (*n* = 4) and 41.19 (±10.47), using the degenerated nerve graft (*n* = 7). All measurements showed no statistical significance (*p* = 0.071) between the groups. Examples of histological samples of retrograde labeled spinal motor neurons are shown in Figure 3.

**TABLE 1:** Spinal motor neuron count in the control and experimental group.

	Control (*n* = 4)	Experimental (*n* = 7)
Spinal motor neurons	66.05 ± 17.03	41.19 ± 10.47
	*p* = 0.071	

### 3.2 Muscle force testing

A comparative analysis was conducted between experimental groups, expressing the recovery as a ratio between the injured and healthy side (Table 2) and a recovery index (RI) (Equation 1) was calculated. A total of 21 animals were operated. In the fresh graft group (*n* = 10), four animals did not show any muscle response, while no measurements were possible in two animals in the experimental group (*n* = 11). For the remaining, the mean MEC in the control animals was 8.2 (±7.1), compared to 8.5 (±4.9) in the predegenerated graft. The mean MT force in the control group was 5.2 (±3.5) compared to 6.4 (±4.4) in the experimental group. Neither the MEC (*p* = 0.462), nor the MT group (*p* = 0.577) showed any significance.

**TABLE 2:** Summary of the MEC and MT (measured in Newton) in the control and experimental group, including the recovery index (RI).

	Control (*n* = 6)			Experimental (*n* = 9)			*p*-value
	Left (N)	Right (N)	RI	Left (N)	Right (N)	RI	
MEC	8.82 (±1.17)	0.75 (±0.66)	8.2 (±7.1)	8.99 (±0.62)	0.76 (±0.47)	8.5 (±4.9)	*p* = 0.462
MT	3.56 (±0.23)	0.18 (±0.13)	5.2 (±3.5)	3.66 (±0.35)	0.24 (±0.17)	6.4 (±4.4)	*p* = 0.577

### 3.3 Muscle weight

**FIGURE 4:**
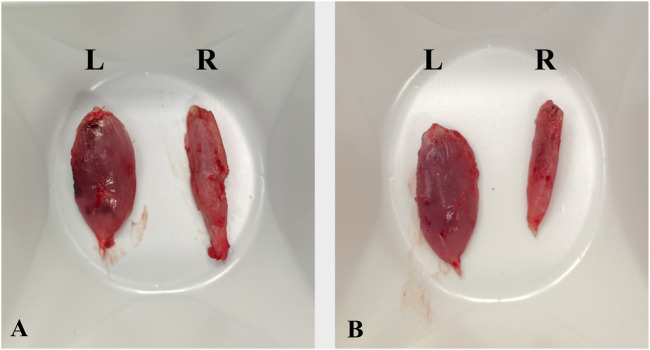
Sample images of the right and left TA in an animal with a fresh **(A)** and a degenerated **(B)** nerve graft. L, left side; R, right side.

The mean weight of the tibialis anterior muscle, comparing the injured to the uninjured side was 0.32 (±0.06) in the control group and 0.32 (±0.04) in the experimental group, with a *p* = 0.964 showing no significance. The results are expressed as a weight ratio between the right (operated) and left (non-operated) side (Table 3). Examples of TA of both subgroups are shown in Figure 4.

**TABLE 3:** Summary of the muscle weight in the control and the experimental animals.

	Control (*n* = 10)		Experimental (*n* = 11)	
	Left	Right	Left	Right
Muscle weight (mg)	1251.0 (±91.1)	402.0 (±88.04)	1191.82 (±114.44)	382.73 (±58.15)
Weight ratio	0.32 (±0.06)		0.32 (±0.04)	
	*p* = 0.964			

## 4 Discussion

Reconstructing BP injuries is surgically challenging, and the clinical outcomes are moderate even with modern developments in surgical techniques and the increased understanding of physiological changes following nerve injuries (Fox and Mackinnon, 2011; Martin et al., 2019). In cases of extensive brachial plexus injury, sensory nerves like the medial antebrachial cutaneous nerve, along with other sensory or mixed sensorimotor nerves, may also be affected and there is a dispute whether these can support neural regeneration over longer distances, since they lack the necessary cellular support.

Among the available surgical options, autologous nerve grafting is regarded as the clinical gold standard. However, this approach is associated with sensory loss and carries a risk of other donor-site complications (Martin et al., 2019). Especially in pan-plexopathies, where fresh donor nerves are limited, other options for nerve harvesting should be explored.

In 1928, Cajal recognized the potential of degenerated nerves, as the neural pathways have been cleared from disintegrating myelin and other structural protein debris (Cajal, 1928). Further on, Gordon et al. investigated the potential of fresh grafts and grafts which have been degenerated over 2 weeks and the use of those in either fresh or degenerated lesions in a rat model (Gordon et al., 1979). The authors compared muscle tension and axonal growth 40 days after nerve reconstruction and noticed that fresh grafts do better in fresh lesions and degenerated nerve grafts in degenerated lesions, which partially corresponds with our findings.

Gulati et al. also investigated the idea of the regeneration potential of degenerated nerve grafts of different age (Gulati, 1996; Gomez-Sanchez et al., 2015). In their publication, the regenerative potential of a graft, was non-inferior for a degeneration time between 6 weeks and 3 months, while grafts degenerated for 6 or even 12 months seem to have less regenerative potential in a fresh lesion. These findings were confirmed by others in regards to the potential of muscle regeneration (Fu and Gordon, 1995). More recent findings indicate that shorter periods can also be effective. Kerns et al. for instance performed a similar experimental animal study using fresh and pre-degenerated nerve grafts in either a fresh or degenerated lesion using a 7-day degeneration period (Kerns et al., 1993). Whilst they successfully showed that both types of nerves grafts had similar regrowth velocities, with regeneration onset in degenerated grafts occurring earlier, there might be valid arguments to extend the degeneration time. The 3-week period we chose in our study might be beneficial to allow completion of the Wallerian degeneration. This method allows more time for Schwann cells to proliferate and align, enhancing neurotrophic support and creating clearer pathways for axonal regrowth (Gaudet et al., 2011). Compared to a 7-day period, this extended timeframe should promote better clearance of cellular debris and ECM remodeling, fostering a more supportive environment for regeneration (Gaudet et al., 2011). Other preclinical studies utilizing degenerated nerve grafts have demonstrated after 8 days, that mid-graft sections of degenerated nerve grafts exhibited a greater number of neurofilaments, indicating a more advanced state of Wallerian degeneration, which correlated with an increased presence of Schwann cells (Kerns et al., 1993). Additionally, after 4 weeks, the degenerated nerve grafts still showed a significant accumulation of debris, with only a few remaining axons detected (Dubuisson et al., 1997).

Further comparison between different degeneration periods on a cellular level might provide clearer recommendations for clinical use.

After peripheral nerve injury, adult Schwann cell (SC), de-differentiate into repair SC, which fulfill critical functions in peripheral nerve regeneration. This includes the phagocytic clearance of myelin debris, the recruitment of macrophages, the organization of Büngner bands to facilitate axonal guidance, and the upregulation of cell surface molecules and trophic factors (Gomez-Sanchez et al., 2017; Jessen and Mirsky, 2019; Jessen et al., 2015; Jessen and Mirsky, 2016; Jang et al., 2016).

Therefore, SC and macrophages have an important role during the Wallerian degeneration and benefiting it Tomita et al. (2009) and Sulaiman and Gordon (2013). This reorganization typically spans 2–3 weeks, an interval that does not need to be traversed when employing a degenerated nerve graft, thereby allowing for immediate regeneration (Gaudet et al., 2011). Our study supports claims that recovery seems to set in even faster using a degenerated nerve graft, but this effect levels within the following 6–9 months of follow-up, as suggested by long-term results (Dubuisson et al., 1997; Bertelli et al., 2006).

As noted, the use of degenerated nerve grafts for reconstructive purposes has been extensively studied, predominantly using rodent sciatic nerve models, which underscores the reliability and suitability of our experimental setup (Wood et al., 2011). In our study, we chose key variables such as muscle weight and motor neuron counts, as these metrics are widely recognized in the literature for assessing nerve reconstruction outcomes and are thus considered robust indicators of regenerative success (Wood et al., 2011). To be able to compare the functionality in each individual animal, we compared the reconstructed to the non-reconstructed side.

Therefore, we focused on the reconstruction of a large proximal nerve defect similar to a plexus lesion, investigating the potential of degenerated nerve grafts in this kind of degenerated nerve lesions. Like in a clinical setting we accepted the size mismatch of the CPN and the SN in order to spare nerve grafts, even if this could affect the regeneration potential of the nerve (Aszmann et al., 1997).

Beside the muscle force testing for functionality we focused on the retrograde labeling of spinal motor neurons as the most indicative and robust proof of successful axonal regeneration. To our best knowledge this has not been performed in a CPN model in rodents using a degenerated nerve graft in a degenerated nerve lesion.

As shown in our results, retrograde labeling of the CPN was performed to quantify motor neurons in the spinal cord, 6 weeks post-reconstruction. We successfully labeled the motor neurons of 11 animals and counted 66.05 (±17.03) neurons in the animals of the control subgroup and 41.19 (±10.47) in the experimental subgroup. However, the groups did not, show any significance (*p* = 0.071). In three animals of the control group, the retrograde labeling did not show any spinal motor neurons, which could be due to a technical error. These animals were excluded from the final analysis. Whilst the values of spinal motor neuron count differs to numbers from literature, e.g., studies with non-injured, fresh CPN suggest, that the CPN has approximately 457–632 spinal motor neurons (Swett et al., 1991; Yeong et al., 1998). Although we counted less spinal motor neurons, we think that our results indicate a sufficient, early reinnervation in a relatively short time period. However, future studies might evaluate later stages of reinnervation as well.

It was not possible to perform muscle force testing in the same animals, since FR takes approximately 5–8 days to pass retrograde into the spinal cord, including another surgical procedure in all animals instead of just one group. For that reason, functional testing had to be conducted in another group. Examination of target muscle functionality remains one of the most important and practiced tools in nerve reconstruction (Wood et al., 2011). For that reason, we tested the MEC and MT of the TA in each animal bilateral in the second study group. In total, six animals, two from the experimental and four from the control group, were excluded from analysis due to missing muscle response while muscle force testing. Comparable to Group A, a higher number of animals in the experimental arm showed a competent muscle function compared to the control group. The MEC was 8.2 (±7.1) in the fresh and 8.5 (±4.9) in the degenerated group and did not show any significance (*p* = 0.462). The MT was 5.2 (±3.5) using a fresh nerve graft and 6.4 (±4.4) using the degenerated nerve graft. The groups did not show a significant difference (*p* = 0.577). Earlier muscle reinnervation might be an explanation for this advantage in the short follow-up period.

According to our opinion, in a clinical setting, it is crucial to evaluate the extend of plexus injuries early and to evaluate the potential for recovery. We believe that in specific cases, the use of degenerated nerve grafts may be beneficial, as preclinical studies have demonstrated their ability to facilitate faster recovery.

Our study has shown, that in an experimental setting, using a degenerated nerve graft in a degenerated lesion is feasible and not inferior to a fresh nerve graft. So far, there is no evidence in the literature, that degenerated nerves have been used to reconstruct nervous lesions in patients, e.g., injuries of the PB, where nerve grafts are rare.

## 5 Conclusion

Our observations suggest that degenerated nerve grafts provide a reliable scaffold for robust nerve regeneration for large defects over long distances and can thus serve as a potential donor reservoir. Most patients with brachial plexus injuries present to tertiary centers between 6 weeks and 6 months after injury. This is a crucial, if a sural nerve graft is not sufficient, and other nerve grafts from the affected extremity could be easier to harvest. This work provides robust evidence, that degenerated nerve grafts provide a similar quality of nerve regeneration and outcome as compared to fresh nerve grafts and could therefore be considered in a reconstructive procedure. We do acknowledge, that experimental data in rodents does not directly translate to humans. However, previous clinical work of our and other centers indicates that indeed both types of grafts provide solid axonal regeneration.

## Data Availability

The original contributions presented in the study are included in the article/supplementary material, further inquiries can be directed to the corresponding author.
